# Identification of Novel Candidate Markers of Type 2 Diabetes and Obesity in Russia by Exome Sequencing with a Limited Sample Size

**DOI:** 10.3390/genes9080415

**Published:** 2018-08-17

**Authors:** Yury A. Barbitoff, Elena A. Serebryakova, Yulia A. Nasykhova, Alexander V. Predeus, Dmitrii E. Polev, Anna R. Shuvalova, Evgenii V. Vasiliev, Stanislav P. Urazov, Andrey M. Sarana, Sergey G. Scherbak, Dmitrii V. Gladyshev, Maria S. Pokrovskaya, Oksana V. Sivakova, Aleksey N. Meshkov, Oxana M. Drapkina, Oleg S. Glotov, Andrey S. Glotov

**Affiliations:** 1 Biobank of the Research Park, Saint Petersburg State University, 199034 Saint Petersburg, Russia; barbitoff@bioinf.me (Y.A.B.); e.a.zhukova@spbu.ru (E.A.S.); yulnasa@gmail.com (Y.A.N.); dmitrii.polev@spbu.ru (D.E.P.); shuvalova_anna_radionovna@mail.ru (A.R.S.); 2 Bioinformatics Institute, 194100 Saint Petersburg, Russia; predeus@bioinf.me; 3 Department of Genetics and Biotechnology, Saint Petersburg State University, 199034 Saint Petersburg, Russia; 4 Institute of Translation Biomedicine, Saint Petersburg State University, 199034 Saint Petersburg, Russia; asarana@mail.ru (A.M.S.); sgsherbak@mail.ru (S.G.S.); 5 Laboratory of Prenatal Diagnostics of Hereditary Diseases, FSBSI «The Research Institute of Obstetrics, Gynaecology and Reproductology Named after D.O. Ott», 199034 Saint Petersburg, Russia; olglotov@mail.ru; 6 City Hospital No. 40, Sestroretsk, 197706 Saint Petersburg, Russia; Vasilievev@mail.ru (E.V.V.); urasta@list.ru (S.P.U.); gladyshev@gmail.com (D.V.G.); 7 Federal State Institution «National Medical Research Center for Preventive Medicine» of the Ministry of Healthcare of the Russian Federation, 101990 Moscow, Russia; mpokrovskaya@gnicpm.ru (M.S.P.); osivakova@gnicpm.ru (O.V.S.); meshkov@lipidclinic.ru (A.N.M.); odrapkina@gnicpm.ru (O.M.D.)

**Keywords:** type 2 diabetes, obesity, next-generation sequencing, exome sequencing, susceptibility locus, single nucleotide polymorphisms, association study

## Abstract

Type 2 diabetes (T2D) and obesity are common chronic disorders with multifactorial etiology. In our study, we performed an exome sequencing analysis of 110 patients of Russian ethnicity together with a multi-perspective approach based on biologically meaningful filtering criteria to detect novel candidate variants and loci for T2D and obesity. We have identified several known single nucleotide polymorphisms (SNPs) as markers for obesity (rs11960429), T2D (rs9379084, rs1126930), and body mass index (BMI) (rs11553746, rs1956549 and rs7195386) (*p* < 0.05). We show that a method based on scoring of case-specific variants together with selection of protein-altering variants can allow for the interrogation of novel and known candidate markers of T2D and obesity in small samples. Using this method, we identified rs328 in *LPL* (*p* = 0.023), rs11863726 in *HBQ1* (*p* = 8 × 10^−5^), rs112984085 in *VAV3* (*p* = 4.8 × 10^−4^) for T2D and obesity, rs6271 in *DBH* (*p* = 0.043), rs62618693 in *QSER1* (*p* = 0.021), rs61758785 in *RAD51B* (*p* = 1.7 × 10^−4^), rs34042554 in *PCDHA1* (*p* = 1 × 10^−4^), and rs144183813 in *PLEKHA5* (*p* = 1.7 × 10^−4^) for obesity; and rs9379084 in *RREB1* (*p* = 0.042), rs2233984 in *C6orf15* (*p* = 0.030), rs61737764 in *ITGB6* (*p* = 0.035), rs17801742 in *COL2A1* (*p* = 8.5 × 10^−5^), and rs685523 in *ADAMTS13* (*p* = 1 × 10^−6^) for T2D as important susceptibility loci in Russian population. Our results demonstrate the effectiveness of whole exome sequencing (WES) technologies for searching for novel markers of multifactorial diseases in cohorts of limited size in poorly studied populations.

## 1. Introduction

Type 2 diabetes (T2D) and obesity are common chronic disorders with multifactorial etiology. Due to their rising prevalence, they are recognized as a global epidemic by the World Health Organization (WHO) [1,2,3,4]. Clinically, T2D is a metabolic disorder characterized by insulin resistance and pancreatic β-cell dysfunction because of uncontrolled hyperglycemia [5]. T2D can seriously impair overall quality of life and lead to a long list of complications, including heart disease, stroke, kidney failure, neuropathy, blindness, and extremity amputation [4,6]. Obesity is a heterogeneous disorder that can be defined as abnormal or excessive fat accumulation that may impair health. Excessive weight and obesity are strongly correlated with T2D. They can lead to adverse metabolic effects on blood pressure, cholesterol, triglycerides and insulin resistance. According to WHO, excessive weight and obesity account for 44% of diabetes cases [7,8]. Because of this close relationship, T2D and obesity can be considered as associated pathologies with a potential common genetic component.

In the recent years the understanding of etiology of these disorders has improved dramatically and genetic high-resolution technologies allowed the identification of 128 susceptibility genetic markers of T2D and more than 700 markers for increased body mass and obesity [4,9,10,11]. The genetic architecture of T2D and obesity was elucidated mostly using genotyping array data to conduct large-scale genome wide association studies (GWAS) [9,10,11]. Despite having high statistical power to detect genetic associations, GWAS-derived single nucleotide polymorphisms (SNPs) themselves do not usually have any impact on complex traits; rather, they are in high linkage disequilibrium (LD) with the real causal variants for the disease. Such causal variants must be finely mapped using imputation methods before making any functional assumptions. Importantly, many complex traits are shaped by a complex interplay between common and rare variants, with the latter usually being missed by conventional GWAS approaches.

Next-generation sequencing (NGS) technologies have become an important instrument in identifying genetic causes of monogenic diseases. Advances in the application of this method are due to two reasons: Reduction in the cost of analysis and development of effective bioinformatics methods. It has been shown that the exome sequencing allowed to identify up to 42% causes of monogenic and oligogenic diseases [12]. However, despite the rapid spread of NGS methods in diagnosis of hereditary diseases, the cost-efficiency of whole-exome and whole-genome sequencing technologies for unveiling the risk factors of complex diseases is still being debated. Exome sequencing of large cohorts has provided important insights into the role of rare coding variants in T2D and obesity [13,14]. However, whole exome sequencing (WES)-based association studies usually suffer from sample size limitations, as thousands of sequenced individuals are usually required to detect exome-wide significant loci, especially for highly polygenic traits. As such, the application of exome sequencing to the analysis of complex traits requires large-scale research efforts and/or development of special methods of bioinformatics analysis. On the other hand, a traditional GWAS approach implying the use of genotyping arrays is cheaper but requires additional studies, such as fine-mapping of causal variants, to get insights into the pathogenesis of complex diseases.

Given these limitations of modern approaches, we developed and applied a multi-perspective approach together with biologically meaningful filtering criteria to detect novel candidate variants and loci for T2D and obesity in a moderate-sized cohort of Russian patients.

## 2. Materials and Methods 

The study was approved by the Review Board of City Hospital No. 40 (Protocol 119, 09.02.2017) Saint-Petersburg and Biobank of Center for Preventive Medicine (Protocol No. 02-05/15, 10.03.2015, and No. 05-05/15, 09.06.2015), Moscow. Written informed consent of the patients was obtained before collecting the samples and processing the medical history data. The study was performed in accordance with the Declaration of Helsinki.

### 2.1. Patients

The study comprised 110 participants. All patients were interviewed to obtain their family history of T2D mellitus and obesity, as well as ethnicity. Height, weight, and waist and hip circumferences were recorded for each patient, and body mass index (BMI) and waist-hip ratio (WHR) was calculated. Fasting glucose and lipid concentrations in the plasma were measured for each study participant. All patients were divided into three groups: 21 patients with obesity (age 55.76 ± 8.6 years, 61.9% male, BMI 43.05 ± 5.7 kg/m^2^), 49 patients with obesity and T2D (age 50.21 ± 10.82 years, 34.7% male, BMI 32.10 ± 8.92 kg/m^2^) and 40 patients of the control group (age 56.44 ± 10.8 years, 52.5% male, BMI 22.56 ± 1.85 kg/m^2^) who met the inclusion criteria for analysis. The main clinical characteristics of the donors are given in Table 1. All patients were of Russian ethnicity.

### 2.2. DNA Isolation

DNA samples from the blood of all patients were isolated by fenol extraction [15]. DNA concentration was determined using Quantus Fluoremeter™ and QuantiFluor^®^ dsDNA System (Promega Corporation, Madison, Wisconsin, USA). DNA integrity was verified using electrophoresis in 0.6% agarose gel in SB buffer. 

### 2.3. Library Preparation and Exome Sequencing

Libraries were constructed using the TruSeq^®^ Exome Library Prep kit (Illumina Inc., San Diego, California, USA) according to the TruSeq Exome Library Prep Reference Guide, Document # 15059911 v01 (Illumina Inc.). Alternatively, libraries were constructed using NimbleGen SeqCap EZ Med Exome Enrichment Kit (Roche NimbleGen Inc., Wisconsin, USA) according to the SeqCap EZ Library SR User’s Guide v. 5.1. Validation of the libraries was performed on the QIAxcel Advanced System (Qiagen, Hilden, Germany). Library quantification was performed using Quantus Fluoremeter™ and QuantiFluor^®^ dsDNA System (Promega Corporation). Paired-end sequencing of the libraries was performed on HiSeq 4000 System or HiSeq 2500 System (Illumina, Inc.). An average sequencing depth of 50.3 million reads per sample was obtained, resulting in the mean coverage of targeted regions of 57.0 and an average of 90.3% of targeted bases covered at least 10×.

### 2.4. Bioinformatic Analysis

All samples were analyzed using a bioinformatic pipeline based on BWA-MEM v. 0.7.15-r1140 [16], PicardTools v. 2.2.2 (http://broadinstitute.github.io/picard/) and Genome Analysis ToolKit (according to the GATK Best Practices workflow [17,18]). Variants were called using GATK HaplotypeCaller in the emit reference confidence (ERC) GVCF mode. All samples included in the dataset were jointly genotyped with GATK GenotypeGVCFs tool; variant filtration was done using Variant Quality Score Recalibration (VQSR) [17] with stringent filtering criteria (SNP and insertion and deletions (INDEL) sensitivity 90.0). Variant annotation was performed using SnpEff and SnpSift packages [19]. We used dbSNP build 146, ClinVar v. 2018-04-01 and dbNSFP v. 2.9 for variant annotation [20]. Allelic frequency data were obtained from VCF files of 1000 Genomes phase 3 (build 2013-05-02, http://www.internationalgenome.org/) [21], Exome Aggregation Consortium (ExAC) r. 0.3.1 [22], and an in-house database of allelic frequencies in the Saint Petersburg State University (SPBU) Biobank cohort according protocol described earlier [23]. 

### 2.5. Statistical Analysis

To run association tests, we used PLINK/SEQ genetics library v. 0.10 ([24]; https://atgu.mgh.harvard.edu/plinkseq). Given that patients in the dataset were separated into three different groups (control, T2D and non-diabetic obesity), we considered four possible comparisons throughout the binary trait analysis (obesity vs. control, T2D vs. control, T2D vs. obesity + control, and obesity + T2D vs. control). All that called variants were used for binary trait association tests. Coding T2D and obesity markers reported in previous exome sequencing and genotyping array-based studies were selected for replication from [9,10,11,13,14,25]. For the association analysis of quantitative traits (BMI, WHR, glucose and triglyceride concentrations), all study participants were pooled together. We removed extreme outliers for each quantitative trait (i.e., points more than 3 standard deviations away from the group mean). Association tests for quantitative traits were performed using variants with minor allele frequency (MAF) > 0.05 in the dataset. The analyses were adjusted for age and sex of the study participants. For gene-level statistics, we applied burden test for case-unique variants on variants with MAF ≤ 0.05 in the dataset. GENCODE v19 genome annotation (https://www.gencodegenes.org/) was used to conduct gene-level tests. In exome-wide analyses, we used 1 × 10^−6^ as the exome-wide significance threshold for SNP-level tests and 2.5 × 10^−6^ for gene-level tests.

To obtain a list of biologically and statistically justified candidate loci for each binary trait given low statistical power of exome-wide analysis, we considered two groups of variants with the highest potential of being causal for the phenotypes considered (see Results for additional details):

(i) variants in known genes implicated in T2D [10] and obesity [26] with significant effect on the corresponding protein (protein-altering variants) (based on the variant type (only non-synonymous substitutions and coding indels were selected) and pathogenicity prediction (for missense variants) by SIFT [27], PROVEAN [28] and Polyphen2 [29] packages); (significance level of 0.05 was applied for these variants)
(ii) low-frequency variants (MAF between 1% and 10% according to SPBU Biobank data) that are highly specific to the case or control group (see Results for a more detailed analysis of such variants’ properties). (significance level of 0.001 was applied for case-specific variants).

For selection of case- and control-specific variants, we calculated a set of previously described additive scores [30]. The scores were calculated as follows: Score1 = 10 × n_case_ − 50 × n_control_; Score2 = 10 × n_control_ − 50 × n_case_, where n_control_ is the number of genotypes in control group (homo-and heterozygous for alternative allele under the dominant model, and strictly homozygous for the recessive model), and n_case_ is the number of genotypes in case group. To compare the numbers of case- and control-specific variants with the random expectation, we performed in silico sampling experiment in the following way: For each SNP with n non-reference genotypes in a cohort we conducted n Bernoulli trials with the success probability *p* = n_affected_/n. All SNPs with the number of successes (k) k = n were scored as expected case-specific. The sampling was performed 10,000 times to obtain the empirical distributions for the number of group-specific variants.

To obtain estimates of the scoring algorithm performance, we simulated the behavior of variants with 7000 different combinations of parameters (true population MAF (tMAF) ranging from 0.001 to 0.1 and true odds ratio (tOR) ranging from 1 to 7). The simulations were conducted as follows: For each pair of parameters, we calculated MAF in control and diseased subpopulation under selected inheritance model (see supplementary material for detailed description of the procedure). We then made 100,000 random samples of n_case_ diseased and n_control_ control individuals from the virtual Hardy-Weinberg population, with the probability of observing non-reference genotype P = 2AF_controls_ − AF_controls_^2^ for controls and P = 2AF_diseased_ − AF_diseased_^2^ for diseased subsample. The number of non-reference genotypes for each subsample was recorded at each iteration and was used to obtain empirical positive outcome probability (P (Score1 ≥ 20)) and an expected value of additive score for each combination of (tMAF, tOR).

To obtain more accurate estimates of false discovery probability for rare causal variants and account for overrepresentation of such variants in diseased cohort, we calculated MAF-adjusted *p*-value as follows: For each variant under consideration, we ran the sampling procedure described above (with 1,000,000 iterations instead of 100,000) under the tMAF = MAF and tOR = 1 model. The number of samples in which Score1 value was greater than or equal to the observed one was recorded and used to calculate the empirical adjusted *p*-value.

All analyses of genetic association data were performed using R v. 3.4.0 [31]. All code pertinent to statistical analysis can be found at http://github.com/bioinf/diabetes/.

## 3. Results

We first sought to replicate previously established associations of coding genetic variants with T2D, obesity, and quantiatitve traits (BMI, WHR, plasma glucose and triglyceride levels) (Table 2, Section 1). To this end, we selected coding markers significantly associated with these traits from several recent WES-based studies and latest GWAS meta-analyses [9,10,11,13,14,25]. We observed a nominally significant association for only two T2D markers in *RREB1* (Table 2) and *PRKAG1*, one obesity (OB) marker in the *OR2Y1* gene, and for several BMI markers in the *ACP1*, *RBBP6*, and *C14orf39* genes.

We went on to explore the association of exome variants with the binary and quantitative traits considered. No SNPs showed association with both binary and quantitative traits at the exome-wide significance level (Tables S1–S8). Moreover, we observed almost no association trends in the distribution of *p*-value for all phenotypes except BMI (Figures S1 and S2). For the latter one, we identified rs689452 as the strongest associating SNP (Table 2, Section 2). This variant is located in the *NQO1* gene that is a member of the NAD(P)H dehydrogenase (quinone) family and encodes a cytoplasmic 2-electron reductase. This enzyme was shown to have a crucial role in protection against oxidative stress [32]. Furthermore, the rs689452 variant in *NQO1* is significantly associated with height according to the genetic investigation of anthropometric trait (GIANT) consortium [11,33].

**Figure 1:**
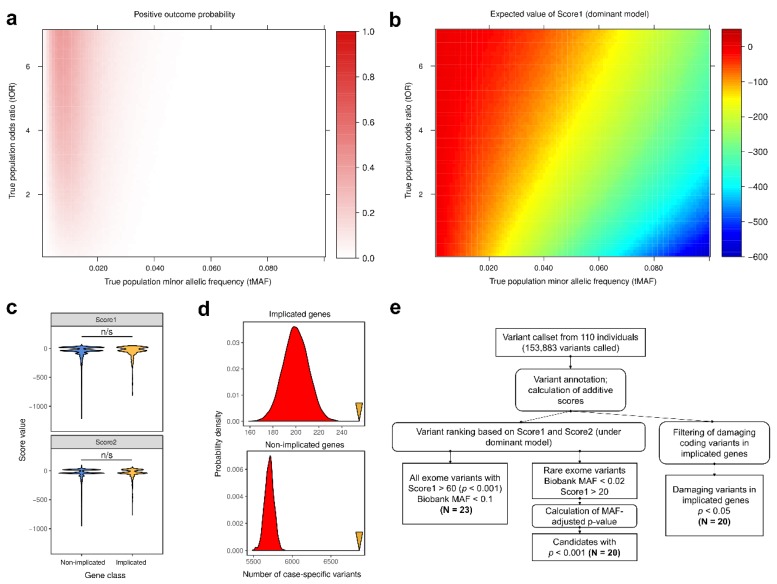
Usage of the scoring and filtering approaches to identify candidate markers of type 2 diabetes (T2D) and obesity in Russian population. (**a**,**b**). Probability of positive test outcome (Score1 > 10) (a) and the expected value of Score1 (b) for variants with different true population minor allele frequency (MAF) and true odds ratio (tOR), as estimated by in silico simulation (see Methods). (**c**). Distribution of values of two additive scores (Score1 and Score2, see text) under dominant inheritance model for damaging coding variants inside genes implicated in T2D and other genes n/s—non-significant difference in *U*-test. (**d**). Distributions of the random expectation numbers of case-unique protein-altering variants inside implicated (top) and non-implicated (bottom) genes. Yellow arrowheads indicate observed values. (**e**). Schematic representation of whole exome sequencing (WES) data analysis used in the present study. Rounded rectangles represent data manipulations.

Given very small number of markers discovered using variant-level tests, we investigated the contribution of rare variants (MAF < 5%) using gene-level burden test for case-unique variants. We observed no genome-wide significant loci with this test, implying that the sample size is insufficient for both SNP-level and gene-level association tests (Figure S3). As both conventional common variant (CVA) and rare variants (RVA) association approaches are drastically underpowered to detect associations in small samples even for quantitative traits, we sought an efficient method to prioritize candidate marker variants and identify biologically and statistically justified candidates for further thorough investigation. We decided to make additional further filtration and ranking of variants to obtain lists of variants which are the most likely candidate markers of the phenotypes concerned. Filtering and scoring approach has shown its efficiency to detect potential risk variants in other diseases such as cardiomyopathy in Russian population [30]. In this work we used two previously described additive scoring approaches to select for case-specific (Score1) and control-specific (Score2) variants under dominant and recessive inheritance models (see Statistical analysis section in the Methods for more details). We first examined the power of such scoring to identify variants with different combinations of MAF and OR. To this end, we conducted in silico simulations for 7000 different combinations of MAF and OR (see Statistical analysis section in the Methods for algorithm details). Our analysis showed that scoring for case-specific variants under dominant inheritance model efficiently prioritizes variants that have low allele frequencies and higher OR, i.e., the ones that have the highest causal potential for the disease. Such variants have both higher probability of positive test outcome (two or more case-specific observations) and higher expected values of case-specificity score (Figure 1a,b). We then went on to compare the distribution of case- and control-specificity scores for protein-altering variants inside genes implicated in T2D/obesity and inside all other genes. Surprisingly, we observed no significant difference in the distributions of both Score1 and Score2 (Figure 1c). However, for both variant groups the number of variants with positive Score1 values (i.e., case-specific ones) is much greater than expected by chance (Figure 1d, OR = 1.44/1.31 (implicated/non-implicated); empirical *p*-value < 0.001). On the other hand, the number of variants specific to the control group (i.e., with positive Score2 values) was lower in both variant classes (Figure S4). These results indicate that while there is no significant enrichment of case-specific variants inside implicated genes compared to the other ones, protein-altering exome variants are overrepresented in the case group and underrepresented in the control one. Hence, we further focused both on two groups of variants with the highest causal potential: (i) protein-altering variants inside known T2D and obesity genes; and (ii) highly case-specific variants with low alternative allele frequency (Figure 1e).

We discovered several reasonable candidate markers of T2D and obesity in Russia inside known T2D and obesity genes (Table 2, Section 3; see Methods). Among these we found rs328 in the *LPL* gene, coding for lipoprotein lipase, associated with T2D and obesity simultaneously (Table 2). The minor allele of rs328 was previously shown to be associated with elevated LDL and decreased HDL; moreover, a recent fine-mapping study suggested its role in the pathogenesis of T2D [34]. We also identified rs6271 in the *DBH* gene, and rs62618693 in the *QSER1* gene as specific markers for obesity. *DBH* is a gene encoding a dopamine β-hydroxylase (DβH) that catalyzes the conversion of dopamine to norepinephrine, which functions both as a hormone and as the main neurotransmitter of the sympathetic nervous system. Earlier it was shown that the rs6271 polymorphic variant affects the plasma DβH activity [35]. This variant has been recently implicated in the regulation of blood pressure levels [36]. Importantly, the rs62618693 variant in *QSER1* has also been recently discovered as a T2D marker using fine-mapping of coding variants [34]. Only one variant inside known causal genes (rs2233984 in the *C6orf15* gene) was identified as a specific T2D marker when comparing T2D and control groups. This variant is also significantly associated with height [33]. However, three additional marker variants (rs9379084 in *RREB1*, rs61737764 in *ITGB6*, and rs17801742 in *COL2A1*) were discovered when comparing T2D patients over control and obese groups together. The *RREB1* gene encodes a transcription factor that binds to RAS-responsive elements (RREs) of gene promoters. Earlier it was demonstrated that RREB-1 exerts a repressive activity on the HLA-G and it was also described as a coactivator of calcitonin, c-erbB2, and secretin genes [37,38,39,40]. Recent studies have shown the association of variant rs9379084 of *RREB1* gene with fat distribution, fasting glucose [41,42], and strong association with T2D [10,34,43]. *ITGB6* gene encodes an integrin β-6 that is a transmembrane glycoprotein receptors. The rs61737764 variant in *ITGB6* has not been described as a T2D marker; however, it is in modest LD with another previously described non-coding T2D variant, rs7593730.

We then turned to investigate another class of likely causal variants, i.e., the case-specific ones (Table 2, Section 4). We first focused on variants that are rare in the population (SPBU MAF < 0.02) as the major class prioritized by our scoring method (Figure 1). We designed a statistical approach to more accurately estimate the significance of case-specific variant association by calculating the empirical probability of the case-specificity score given known MAF in the population, P (Score1 ≥ N|MAF) (see Statistical analysis section in the Methods). Out of rare case-specific variants, we discovered rs139972217 in *TMC8*, rs61758785 in *RAD51B*, rs34042554 in *PCDHA1*, and rs144183813 in *PLEKHA5* as the most significant candidates (*p_adj_* < 0.001). The *TMC8* gene encodes for a transmembrane protein, playing a role in diverse skin diseases. Variants at the *TMC6*-*TMC8* locus have been associated with the levels of glycated haemoglobin (HbA_1c_), a common biomarker that is used for diagnostics of T2D [44]. Expression levels of another gene harboring association signal, *PLEKHA5*, are linked to seroconversion behind type 1 diabetes [45]. These data indicate potential high relevance of the identified variants for pathogenesis of T2D and obesity.

Out of exome variants with intermediate frequency (0.02 < SPBU MAF < 0.1) with high case-specificity score and statistical support, we found rs11863726 in *HBQ1* and rs112984085 in *VAV3* which were associated with T2D and obesity compared to controls, and rs685523 in *ADAMTS13* as a specific marker for T2D. *HBQ1* gene encodes the hemoglobin subunit theta 1 that is expressed only in human fetal erythroid tissue. The function of this gene is poorly understood. No association for polymorphism of *HBQ1* gene with T2D or other endocrine disorders has been described previously. The gene of guanine nucleotide exchange factors *VAV3* is a member of the VAV family of proto-oncogenes. *VAV3* gene has an impact on angiogenesis, cytoskeleton organization and function, regulation of immune system which renders it a potentially relevant gene for molecular pathology behind T2D [46]. The *ADAMTS13* gene codes a multimeric plasma glycoprotein that plays a critical role in platelet adhesion and aggregation on vascular lesions. Previously it was shown that the circulating von Willebrand factor (VWF) concentrations are elevated in T2D patients, and long-term studies of T2D patients have linked VWF to the development of both microvascular and macrovascular disease [47,48,49]. VWF has been found to be a risk marker for death in T2D [50,51]. The mechanisms behind elevated VWF concentrations in T2D remain unclear, however, these facts imply a potential role of *ADAMTS13* in the pathogenesis of the disease. Overall, all three genes described above seem as relevant targets for further genetic and mechanistic studies.

## 4. Discussion

Type 2 diabetes is one of the severe chronic endocrine diseases, whose prevalence is steadily increasing worldwide [1,2,3,4]. Obesity is strongly correlated with T2D and increases the odds of development of T2D; however, both shared and unique genetic and environmental factors shape the risk of T2D and obesity. Large-scale genetic studies have identified numerous loci associated with T2D and obesity, with the most recent efforts involving hundreds of thousands of individuals of various ancestry [9,10,11,34]. However, genetic variants affecting the risk of a disease in less studied population groups might substantially differ from the ones observed in these studies. Hence, additional genetic studies in these populations are needed to identify common and specific disease markers (exemplified in [52]). 

In our study we applied a multi-perspective approach to identify candidate markers of T2D and obesity in a cohort of Russian patients with T2D and obesity. We applied both conventional SNP-level and gene-level association tests, as well as novel strategies to identify variants associated with T2D, obesity, and relevant quantitative traits (BMI, WHR, glucose, and triglyceride concentration). We present an efficient approach to search for novel candidate markers in WES data that are applicable in smaller samples. This approach is based on rational filtering of protein-altering genetic variants and prioritization of case- or control-specific genetic variants, i.e., the ones with the highest potential of being causal for the disease. We show that, while this approach has low power to identify common variants with low OR, it efficiently prioritizes variants of intermediate and low frequency with higher OR (Figure 1a,b). In order to decrease false discovery probability, which is quite substantial without any additional filters (P (Score ≥ 20) = 0.047 for a variant with MAF = 0.02 and OR = 1), we also apply additional *p*-value adjustments that allow for selection of statistically justified rare variants with low or moderate type 1 error probability.

The application of our approach allowed us to identify potential common and specific markers for diabetes and obesity. We present evidence for potential association of variants in *RREB1, ITGB6, COL2A1, TMC8* and *ADAMTS13* genes with T2D, and of variants in *HBQ1, LPL,* and *VAV3* with both diabetic and non-diabetic obesity. Importantly, some variants (namely, rs685523 in *ADAMTS13* and rs61737764 in *ITGB6*) were specific to the group of T2D patients. It is probable that these markers control the processes initiated by specific metabolic cascades which are less relevant for non-diabetic obesity. Moreover, we observed candidate association of rs6271 in the *DBH* gene, rs62618693 in the *QSER1* gene, and variants in *PCDHA1*, *RAD51B,* and *PLEKHA5* with non-T2D-linked obesity. It can be assumed that these markers are potentially involved in the development of obesity as an independent condition. However, this hypothesis requires further confirmation.

It is important to note that our analysis strategy allowed us to identify several candidate markers of T2D which have been showed to be significantly associated with the phenotype in one of the most recent fine-mapping studies (e.g., rs328 in *LPL*, rsrs62618693 in *QSER1*, and rs9379084 in *RREB1*) [34]. As such, our strategy based on filtering for protein-altering variants inside implicated genes can enhance identification of candidate coding markers in smaller samples. On the other hand, we observed that protein-altering variants are significantly overrepresented in cases compared to control individuals both inside and outside known disease-relevant genes (Figure 1b). While this result may be at least partially explained by weak genetic linkage which could not be resolved given a small sample size, recent results [34] indicate that numerous coding markers for T2D actually lie outside of known disease genes. Hence, it seems useful to consider damaging variants inside both implicated and non-implicated genes in exome sequencing-based studies. Importantly, most of the rare variants identified by case-specific scoring approach are actually annotated as damaging missense mutations in non-implicated genes, i.e., they belong to the protein-altering class.

In our study, we observed several highly case-specific variants in genes previously not directly linked to T2D and/or obesity (e.g., *TMC8*, *PCDHA1*, *PLEKHA5*, *HBQ1*, *VAV3*, and *ADAMTS13*). While many of these variants lack functional validation and were not reported in other studies, genetic alterations or expression changes of some of the corresponding genes are associated with diabetes-related glycemic traits (e.g., HbA_1c_ levels for *TMC8* [44] and T1D-related seroconversion for *PLEKHA5* [45]). Thus, these genes may be selected as potential candidates for further functional investigations and replication of association results.

## 5. Conclusions

Our study shows that whole exome analysis can serve as a reasonable approach for identifying genetic markers of a complex disease even in limited samples. Using our multi-perspective analysis strategy, we have discovered some reasonable candidate loci and SNPs that might play an important role in the pathogenesis of T2D and obesity in the Russian population. Overall, rational filtering and ranking of potentially causal variants offers a valuable strategy for the candidate disease marker discovery by exome sequencing in poorly studied populations, especially if large-scale genetic studies are lacking. As such, this approach may assist in disease gene identification for polygenic traits.

## Figures and Tables

**Table 1:** Basic characteristics of study participants of Russian ethnicity.

Characteristic	Control Group (n = 40)	Obesity Group (n = 21)	T2D (with Obesity) (n = 49)
Age (years)	56.44 ± 10.8	55.76 ± 8.60	50.21 ± 10.82
Male	52.5%	61.9%	34.7%
Ethnicity	Russian	Russian	Russian
BMI (kg/m^2^)	22.56 ± 1.85	43.05 ± 5.70	32.10 ± 8.92
**Family History (based on the results of the questionnaire)**			
Family history of obesity	no (40/40)	yes (21/21)	yes (49/49)
Family history of T2D	no (40/40)	yes (21/21)	yes (49/49)
Family history of other endocrine pathologies	no (40/40)	yes (21/21)	yes (49/49)

BMI: body mass index; T2D: type 2 diabetes.

**Table 2:** Main markers for obesity and type 2 diabetes (T2D) identified by exome analysis.

Test	Position	rsID	Gene	Effect	*p*	MAF	*p_adj_* *	OR/*β*	Comment
**1. Replication of Association (SNP-Level) with Binary/Quantitative Traits**									
BMI vs. all variants	chr2:272203:C>T	rs11553746	*ACP1*	Missense variant	0.012	0.352	-	3.92	
	chr14:60928201:G>A	rs1956549	*C14orf39*	Intron variant	0.013	0.073	-	2.60	
	chr16:24578458:T>C	rs7195386	*RBBP6*	Splice variant	0.029	0.473	-	−1.24	[11]
OB vs. control	chr5:180166677:G>A	rs11960429	*OR2Y1*	Missense variant	0.043	0.014	-	13.6	
T2D vs. C, T2D vs. OBC	chr6:7231843:G>A	rs9379084	*RREB1*	Missense variant	0.013, 0.042	0.097	-	0.26, 0.35	[10,34]
	chr12:49399132:G>C	rs1126930	*PRKAG1*	Missense variant	0.041, 0.037	0.032		0.09,0.09	[9,25]
2. Association with Quantitative Traits **									
BMI vs. all variants	chr16:69752464:C>G	rs689452	*NQO1*	Intron variant	9 × 10^−6^	0.129	-	2.04	Associated with height [33]
**3. Association with Protein-Altering Variants in Known Genes (0.01 < SPBU MAF < 0.1)**									
OB + T2D vs. control	chr8:19819724:C>G	rs328	*LPL*	Stop gained	0.023	0.065	-	0.26	Recently associated [34]
OB vs. control	chr9:136522274:C>T	rs6271	*DBH*	Missense variant	0.043	0.041	-	13.5	Associated with BP *** [36]
	chr11:32956:C>T	rs62618693	*QSER1*	Missense variant	0.021	0.038	-	10.1	Recently associated [34]
T2D vs. control	chr6:7231843:G>A	rs9379084	*RREB1*	Missense variant	0.013	0.097	-	0.26	Recently associated [34]
	chr6:31079264:C>T	rs2233984	*C6orf15*	Missense variant	0.030	0.067	-	0.31	Associated with height [33]
T2D vs. OBC	chr2:160994293:C>T	rs61737764	*ITGB6*	Missense variant	0.035	0.007	-	12.1	Linked withrs7593730
	chr6:7231843:G>A	rs9379084	*RREB1*	Missense variant	0.042	0.097	-	0.35	
**4. Association with Case-Specific Exome Variants (SPBU MAF < 0.1)**									
OB vs. control	chr5:140168291:T>C	rs34042554	*PCDHA1*	Missense variant	0.003	0.017	1.0 × 10^−4^	25.1	
	chr14:69061228:G>A	rs61758785	*RAD51B*	Missense variant	0.035	0.007	1.7 × 10^−4^	12.6	
	chr12:19408017:G>A	rs144183813	*PLEKHA5*	Missense variant	0.015	0.007	1.7 × 10^−4^	17.9	Gene expression affects T1D ^†^ [45]
T2D vs. OBC	chr12:48376869:T>C	rs17801742	*COL2A1*	Splice variant	3.1 × 10^−4^	0.089	8.5 × 10^−5^	10.4	Associated with height [33]
OB + T2D vs. control	chr16:230578:A>G	rs11863726	*HBQ1*	Splice variant	3.9 × 10^−4^	0.094	8 × 10^−5^	22.8	
	chr1:108119007:G>C	rs112984085	*VAV3*	Intron variant	7.1 × 10^−4^	0.063	4.8 × 10^−4^	21.3	
T2D vs. C, T2D vs. OBC	chr9:136310908:C>T	rs685523	*ADAMTS13*	Missense variant	2.7 × 10^−4^, 1.6 × 10^−5^	0.083	7 × 10^−5^, 1 × 10^−6^	26.0, 38.9	
	chr17:76136994:C>T	rs139972217	*TMC8*	Missense variant	0.032, 0.006	0.009	1.4 × 10^−4^, 9.9 × 10^−5^	11.6, 17.9	Gene associated with HbA_1c_ [44]

C—control group, OBC—obese and controls pooled together; *—adjusted *p*-value for selected variants was obtained by bootstrap test conditioned on true population minor allele frequency (MAF) (see Statistical analysis section in the Methods); **—all individuals were pooled for quantitative trait analysis; ***—BP—blood pressure; ^†^—T1D—type 1 diabetes; OR: odds ratio.

## Supplementary Materials

The following are available online at http://www.mdpi.com/2073-4425/9/8/415/s1. A PDF file containing Supplementary Note and Figures S1–S3. **Supplementary note.** Calculation of allelic frequency in the cases and controls, **Figure S1**. Statistics of SNP-level association with 4 selected quantitative traits. **Figure S2**. Statistics of locus-level association with binary traits in indicted comparisons. **Figure S3**. Statistics of SNP-level multi-phenotype association with all quantitative traits. **Figure S4.** Distributions of the random expectation numbers of control-specific protein-altering variants inside implicated genes. Yellow arrowhead indicates observed values. a ZIP archive containing Supplementary Tables S1–S8. **Table S1**. Association data for exome variants for BMI. **Table S2**. Association data for exome variants for glucose concentration. **Table S3**. Association data for exome variants for OB + T2D vs. Control. **Table S4**. Association data for exome variants for OB vs. Control. **Table S5**. Association data for exome variants for T2D vs. Control. **Table S6**. Association data for exome variants for T2D vs. OB + C. **Table S7**. Association data for exome variants for triglyceride concentration. **Table S8**. Association data for exome variants for WHR.
