# Supplementary material for: Identification of Novel Candidate Markers of Type 2 Diabetes and Obesity in Russia by Exome Sequencing with a Limited Sample Size
Source: Genes (Basel). 2018 Aug 17;9(8):415. doi: 10.3390/genes9080415 (PMC6115942; doi:10.3390/genes9080415)
Supplement: Supplementary file 1 [file genes-09-00415-s001.zip › supplementary_material/Supporting_Figures.pdf]

Article

Identification of novel candidate markers of type 2 diabetes and obesity in Russia by exome sequencing with a limited sample size

Yury A. Barbitoff[1,2,3,4], Elena A. Serebryakova[1,5,6], Yulia A. Nasykhova[1,5], Alexander V. Predeus[2]; Dmitrii E. Polev[1], Anna R. Shuvalova[1], Evgenii V. Vasiliev[6], Stanislav P. Urazov[6], Andrey M. Sarana[4,6], Sergey G. Scherbak[4,6], Maria S. Pokrovskaya[7], Oksana V. Sivakova[7], Aleksey N. Meshkov[7], Oxana M. Drapkina[7], Oleg S. Glotov[5,6], Andrey S. Glotov[1,5*]

[1] Biobank of the Research Park, Saint Petersburg State University, Russia, 199034, St. Petersburg, Universitetskaya nab., 7-9-11

[2] Bioinformatics Institute, Saint Petersburg, Russia, 194100, Kantemirovskaya st., 2A

[3] Department of Genetics and Biotechnology, Saint Petersburg State University, Russia, 199034, St. Petersburg, Universitetskaya nab., 7-9-11

[4] Institute of Translation Biomedicine, Saint Petersburg State University, Russia, 199034, St. Petersburg, Universitetskaya nab., 7-9-11

[5] Laboratory of prenatal diagnostics of hereditary diseases, FSBSI «The Research Institute of Obstetrics, Gynaecology and Reproductology named after D.O. Ott», Russia, 199034, St. Petersburg, Mendeleyevskaya line, 3

[6] City hospital №40, Russia, 197706, St. Petersburg, Sestroretsk, Borisov Str., 9

[7] Federal State Institution «National Medical Research Center for Preventive Medicine» of the Ministry of Healthcare of the Russian Federation, 101990, Moscow, Petroverigskiy lane, bld. 10

For correspondence:

Corresponding author: Director of Biobank of the Research Park, Saint Petersburg State University, Russia, 199034, St. Petersburg, Universitetskaya nab., 7-9-11,

Tel.: + 7-911-783-20-03

E-mail address: anglotov@mail.ru (Andrey Glotov)

Supporting Information

Supplementary note

Calculation of allelic frequency in the cases and controls

To calculate the minor allele frequency in case and control groups given known true population minor allele frequency (tMAF) and true odds ratio (tOR), we first reconstructed the contingency table to obtain proportions of case and control individuals with reference or non-reference genotype:

	Reference genotype	Non-reference genotype	
**Control**	p_{11}	p_{10}	$p_{1.}$
**Diseased**	p_{01}	p_{00}	$p_{0.}$
	$p_{.1}$	$p_{.0}$	

Under the dominant inheritance model, $p_{.1}$ is the frequency of reference-homozygous genotype, i.e. $(1 - tMAF)^2$ under Hardy-Weinberg equilibrium. On the other hand, $p_{1.}$ is equal to $1 - P_D$, where P_D is the disease prevalence (0.08 in all simulations presented in the manuscript).

Given these estimates and tOR, we can use functional properties of the odds ratio to obtain cell probabilityies:

$$p_{11} = \frac{1 + ((1 - P_D) + (1 - tMAF)^2)(tOR - 1) - S}{2(tOR - 1)}$$

$$S = \sqrt{(1 + ((1 - P_D) + (1 - tMAF)^2)(tOR - 1))^2 + 4tOR(1 - tOR)(1 - P_D)(1 - tMAF)^2}$$

Then, the cell probabilities can be calculated as follows:

$$p_{01} = 1 - P_D - p_{11};\ p_{10} = (1 - tMAF)^2 - p_{11};\ p_{00} = 1 - p_{11} - p_{10} - p_{01}$$

Then, non-reference allele frequency can be obtained by subtracting the square root of reference genotype frequency in each subpopulation:

$$MAF_{controls} = 1 - \sqrt{\frac{p_{11}}{p_{11} + p_{10}}}$$

$$MAF_{diseased} = 1 - \sqrt{\frac{p_{01}}{p_{01} + p_{00}}}$$

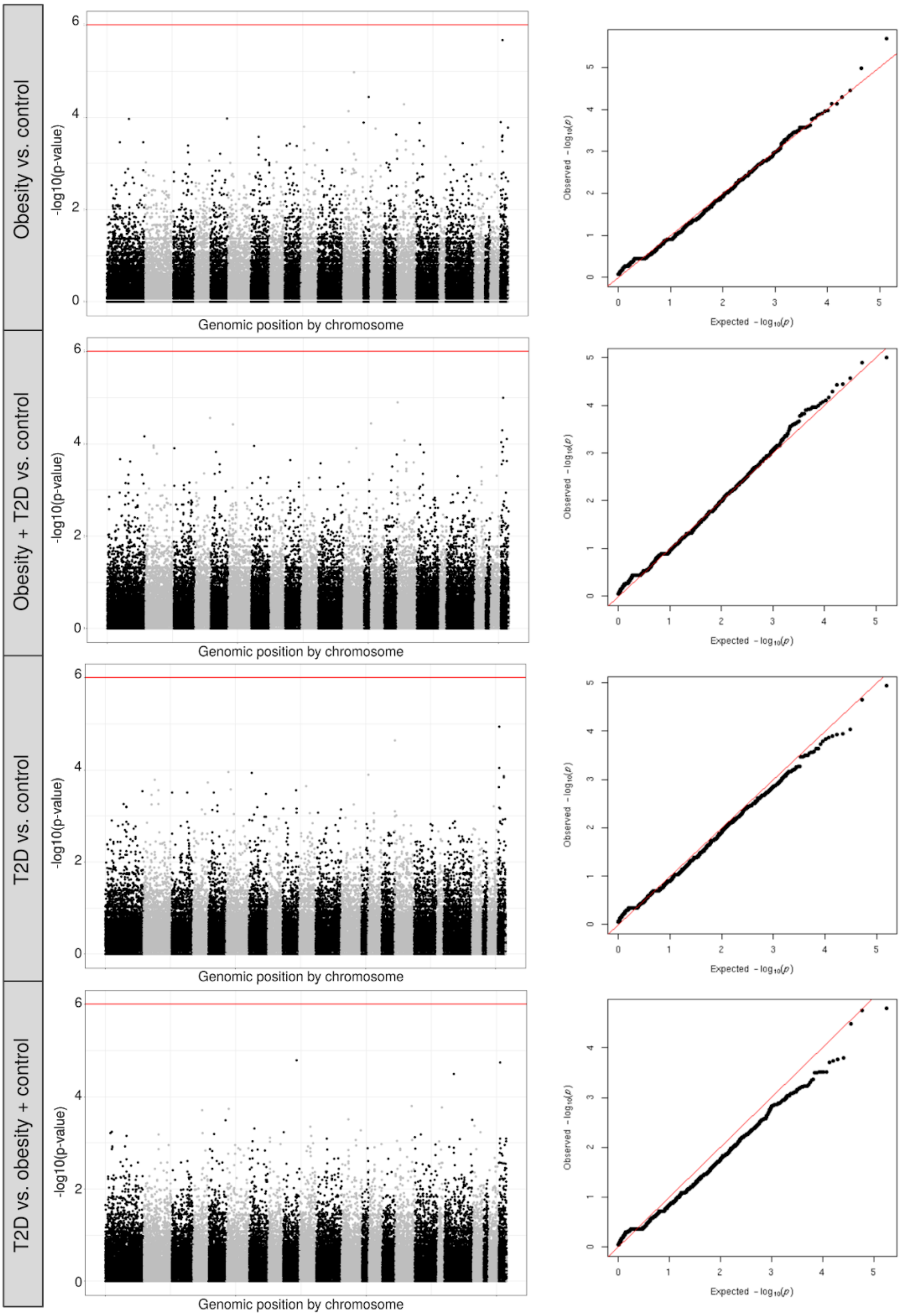

Figure S1. Statistics of SNP-level association in 4 comparisons in binary trati analysis (indicated on the left). Left panels, Manhattan plots of association p-values; right panels, quantile-quantile (Q-Q) plots of p-value distribution.

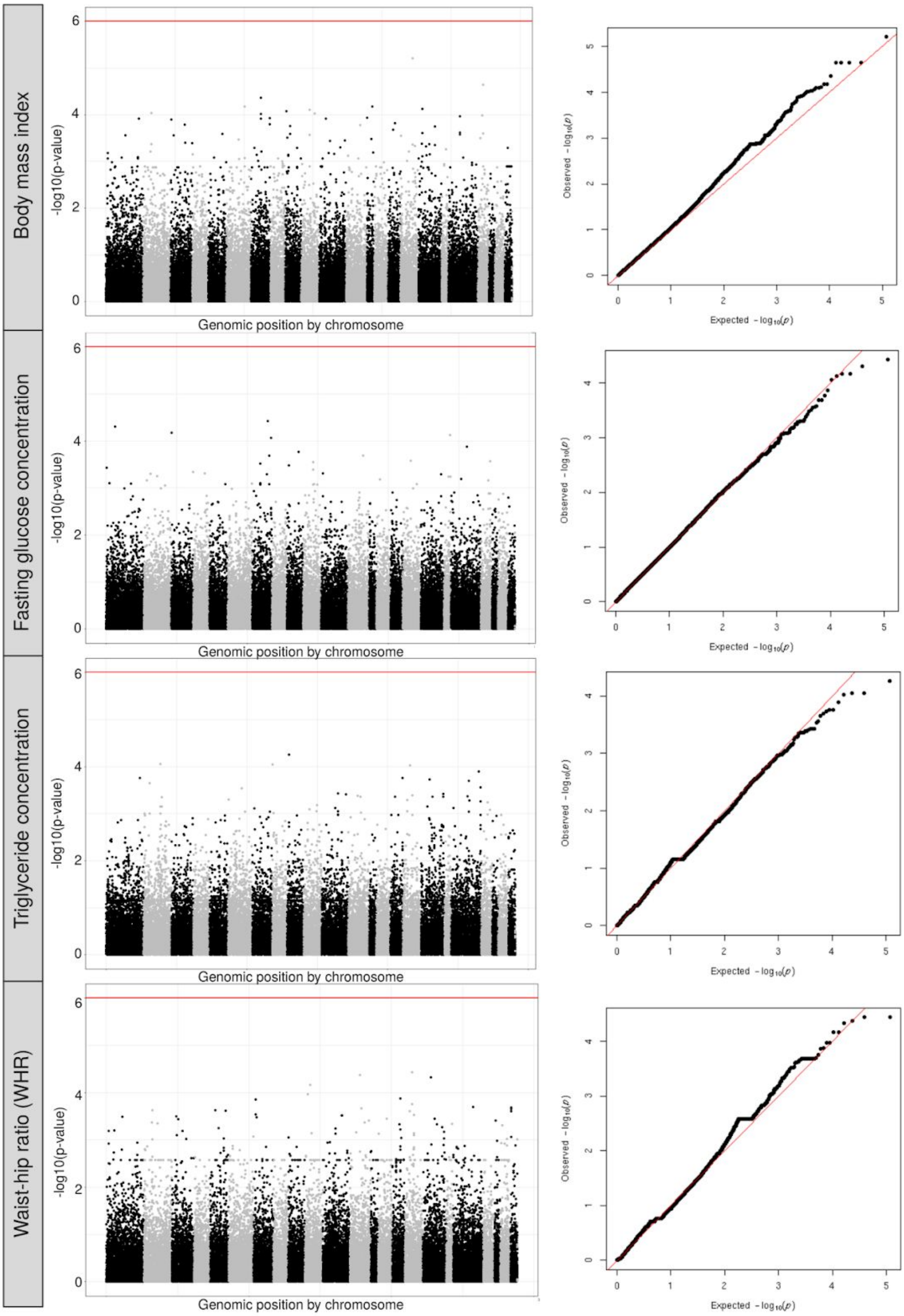

Figure S2. Statistics of SNP-level association with 4 selected quantitative traits (indicated on the left). Left panels, Manhattan plots of association p-values; right panels, quantile-quantile (Q-Q) plots of p-value distribution. Note that inflation of p-value distribution is observed only for body mass index (BMI).

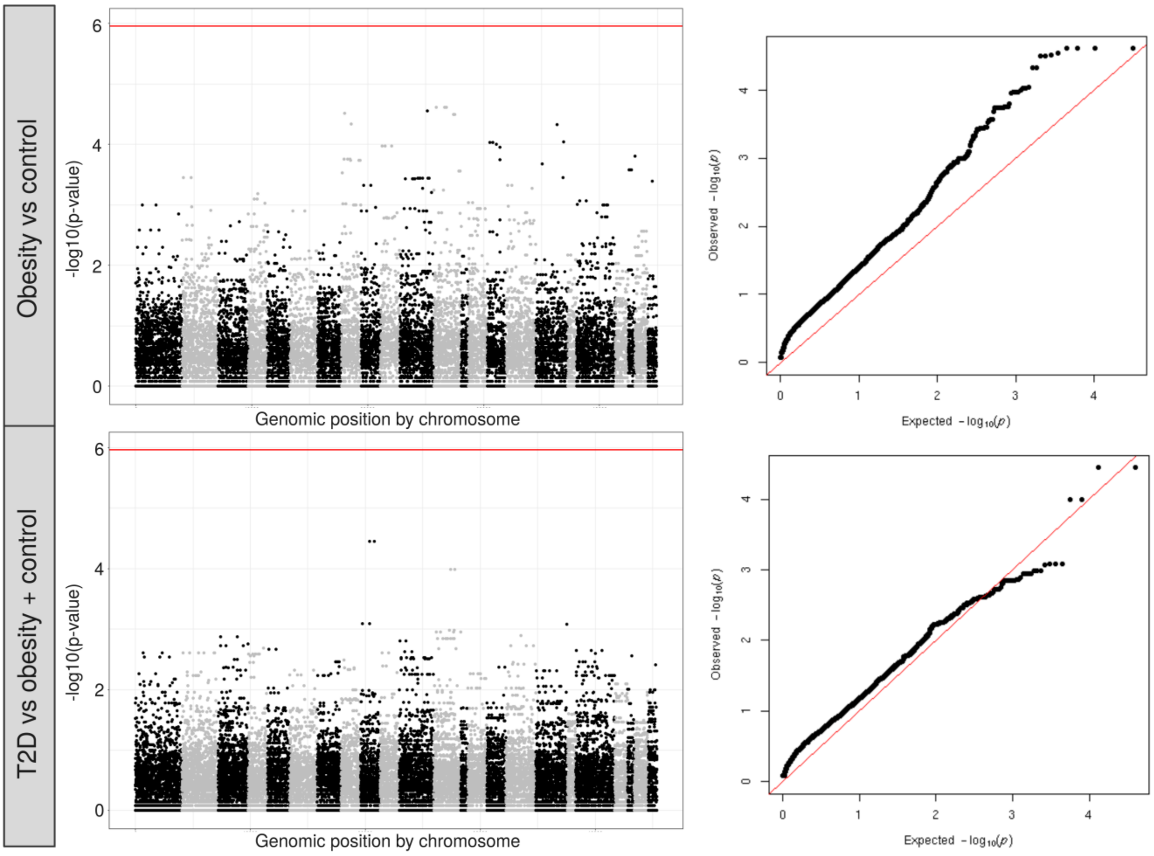

Figure S3. Statistics of locus-level association with binary traits in indicted comparisons. Left panels, Manhattan plots of association p-values; right panels, quantile-quantile (Q-Q) plots of p-value distribution.

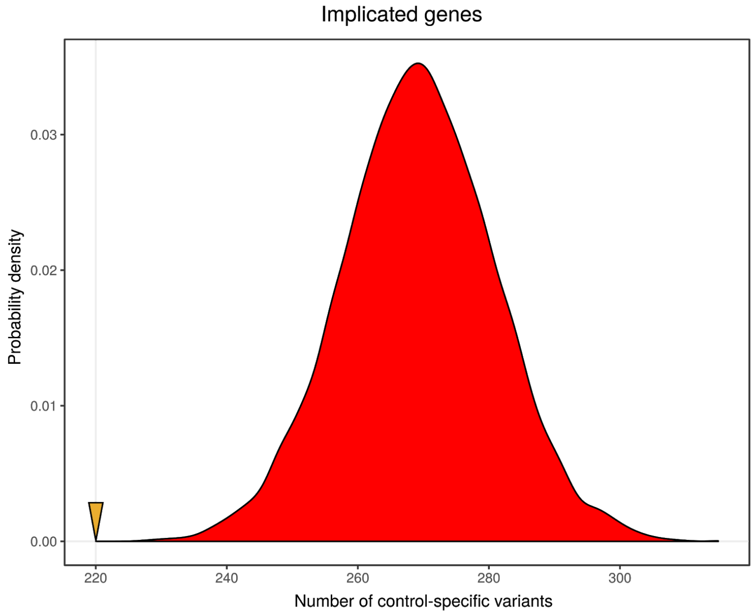

Figure S4. Distributions of the random expectation numbers of control-specific protein-altering variants inside implicated genes. Yellow arrowhead indicates observed values.
